# Microarray gene expression profiling of osteoarthritic bone suggests altered bone remodelling, WNT and transforming growth factor-β/bone morphogenic protein signalling

**DOI:** 10.1186/ar2301

**Published:** 2007-09-27

**Authors:** Blair Hopwood, Anna Tsykin, David M Findlay, Nicola L Fazzalari

**Affiliations:** 1 Division of Tissue Pathology, Institute of Medical & Veterinary Science, Frome Road, Adelaide, South Australia, 5000, Australia; 2 Hanson Institute, Frome Road, Adelaide, South Australia, 5000, Australia; 3 School of Mathematics, University of Adelaide, North Terrace, Adelaide, South Australia, 5005, Australia; 4 Discipline of Orthopaedics & Trauma, University of Adelaide, North Terrace, Adelaide, South Australia, 5005, Australia; 5 Discipline of Pathology, University of Adelaide, North Terrace, Adelaide, South Australia, 5005, Australia

## Abstract

Osteoarthritis (OA) is characterized by alterations to subchondral bone as well as articular cartilage. Changes to bone in OA have also been identified at sites distal to the affected joint, which include increased bone volume fraction and reduced bone mineralization. Altered bone remodelling has been proposed to underlie these bone changes in OA. To investigate the molecular basis for these changes, we performed microarray gene expression profiling of bone obtained at autopsy from individuals with no evidence of joint disease (control) and from individuals undergoing joint replacement surgery for either degenerative hip OA, or fractured neck of femur (osteoporosis [OP]). The OP sample set was included because an inverse association, with respect to bone density, has been observed between OA and the low bone density disease OP. Compugen human 19K-oligo microarray slides were used to compare the gene expression profiles of OA, control and OP bone samples. Four sets of samples were analyzed, comprising 10 OA-control female, 10 OA-control male, 10 OA-OP female and 9 OP-control female sample pairs. Print tip Lowess normalization and Bayesian statistical analyses were carried out using linear models for microarray analysis, which identified 150 differentially expressed genes in OA bone with t scores above 4. Twenty-five of these genes were then confirmed to be differentially expressed (*P *< 0.01) by real-time PCR analysis. A substantial number of the top-ranking differentially expressed genes identified in OA bone are known to play roles in osteoblasts, osteocytes and osteoclasts. Many of these genes are targets of either the WNT (wingless MMTV integration) signalling pathway (*TWIST1*, *IBSP*, *S100A4*, *MMP25*, *RUNX2 *and *CD14*) or the transforming growth factor (TGF)-β/bone morphogenic protein (BMP) signalling pathway (*ADAMTS4*, *ADM*, *MEPE*, *GADD45B*, *COL4A1 *and *FST*). Other differentially expressed genes included WNT (*WNT5B*, *NHERF1*, *CTNNB1 *and *PTEN*) and TGF-β/BMP (*TGFB1*, *SMAD3*, *BMP5 *and *INHBA*) signalling pathway component or modulating genes. In addition a subset of genes involved in osteoclast function (*GSN*, *PTK9*, *VCAM1*, *ITGB2*, *ANXA2*, *GRN*, *PDE4A *and *FOXP1*) was identified as being differentially expressed in OA bone between females and males. Altered expression of these sets of genes suggests altered bone remodelling and may in part explain the sex disparity observed in OA.

## Introduction

Osteoarthritis (OA) is a complex, multifactorial, age-dependent degenerative disease of the synovial joints. It affects the knee and the hip most commonly, and females at a higher rate than males, particularly after the menopause [1]. OA is characterized by changes to all components of the joint, with degeneration and loss of articular cartilage and changes to the subchondral bone being constant factors in disease progression [2]. Along with the breakdown of the cartilage and joint space narrowing, there is thickening and sclerosis of subchondral bone, development of cysts and bony overgrowth at the margins of the joint. Despite an increase in bone volume fraction, the subchondral bone is mechanically weaker in OA because of hypomineralization, increased collagen metabolism and altered bone remodelling [3,4]. Evidence from animal models of OA suggests that the changes in the density and metabolism of subchondral bone develop concomitantly with the signs of cartilage damage [5-7]. In addition, there is now evidence in animal OA models that antiresorptive agents, which inhibit subchondral bone remodelling, also prevent the bone changes and loss of cartilage seen in OA, thus reducing joint damage [8,9]. A human trial of an antiresorptive agent also showed clear trends toward improvement in both joint structure and symptoms in patients with primary knee OA [10]. These findings are consistent with the hypothesis that OA is a bone disease, rather than – or in addition to – a cartilage disease, and that the structural and compositional changes seen in OA subchondral bone, brought about by altered bone remodelling, contribute to the breakdown of the articular cartilage at the joint [11-14].

There is also evidence that the osteoblasts in subchondral bone can influence chondrocyte and cartilage metabolism more directly, leading to abnormal remodelling of OA cartilage [15,16]. In articular joints there is a complex juxtaposition of vascular elements, subchondral bone and the different cartilage layers, with important communication between these tissues [17]. These observations point to a clear interplay between bone and cartilage at articular joints and show that these tissues represent a functional cellular and molecular unit [18]. Altered angiogenesis could also be contributing to the changes seen in OA bone and cartilage, because important inter-relationships between bone remodelling, chondrogenic and angiogenic processes are now emerging [19-21].

In addition to the changes observed in subchondral bone, there is growing evidence for generalized involvement of bone in the pathogenesis of OA. Studies investigating bone at sites distal to the joint cartilage degeneration, such as the intertrochanteric (IT) and medial principal compressive regions of the proximal femur, and the iliac crest, have yielded evidence of altered bone composition and increased bone volume in OA compared with control individuals [22-25]. It has been proposed that these structural and compositional changes reflect systemic differences in OA bone remodelling compared with control bone, and when these changes operate in subchondral bone they can contribute to the breakdown of the articular cartilage and eventual failure of the joint [11-14]. Furthermore, an inverse association between OA and the low bone density disease osteoporosis (OP) has been observed. OA patients rarely proceed to osteoporotic fracture, suggesting that OA has a protective effect on progression of OP. Conversely, OA is reported to be rare in OP individuals [26].

The structural and compositional changes seen in OA bone are likely to have considerable genetic input because there is a significant heritable component to OA, as judged by genetic studies [27]. Interestingly, many of the candidate susceptibility genes for OA identified by genetic screening approaches have bone-related functions, further suggesting the involvement of bone in OA. Primary OA candidate genes identified, with bone-related functions, include *COL1A1*, *VDR*, *ESR1*, *IGF1*, *SFRP3*, *BMP5 *and *TGFB1 *[27-30]. *SFRP3 *encodes a decoy receptor for WNT (wingless MMTV integration) ligands and plays a role in osteoblast differentiation [31]. The WNT signalling pathway is a major developmental pathway that is involved in cell fate, differentiation and proliferation. This signalling pathway has also been linked to skeletal development and bone pathologies such as OP [32]. The identification of *TGFB1 *and *BMP5*, a member of the transforming growth factor (TGF)-β superfamily, as OA susceptibility loci has implicated the TGF-β/BMP signalling pathway in OA pathogenesis. The TGF-β/BMP signalling pathway plays important roles in development, cell proliferation and differentiation, and it has also been shown to influence bone mass and bone remodelling [33,34].

Complementing the human genetic studies described above, and in support of altered bone remodelling at sites distal to the active subchondral disease site, we previously identified differences in the expression of known skeletally active genes in human trabecular bone obtained from the IT region from individuals with hip OA, as compared with bone from the same site in control individuals. Genes identified as differentially expressed include downregulated osteoclastogenic factor genes (*RANKL*, *RANK*, *IL6 *and *IL11*) and upregulated bone formation marker genes (*ALPL*, *BGLAP*, *SPP1 *and *COL1A2*) [35-37]. Others have identified in OA individuals altered levels of insulin-like growth factor-1, insulin-like growth factor-2 and TGF-β_1 _in cortical bone from the iliac crest [38]; matrix metalloproteinase (MMP)2 and liver alkaline phosphatase in subchondral bone [4]; and IL-1β, IL-6 and TGF-β_1 _in human primary subchondral osteoblasts [39].

In the present study, we used microarray analysis to survey comprehensively the expression levels of many thousands of genes simultaneously in trabecular bone from the IT region of the proximal femur and to compare gene expression in bone from OA, control and OP individuals. We identified altered expression of WNT and TGF-β/BMP signalling pathway and target genes in OA bone. The genes include those with known or suspected roles in osteoblast, osteocyte and osteoclast differentiation and function, supporting a role for altered bone remodelling in OA pathogenesis.

## Materials and methods

### Human bone samples

For the OA and OP groups, tube saw bone biopsies (10 mm diameter and 20 to 40 mm long) were obtained from the IT region of the proximal femur. These were obtained from 24 patients (14 females [age range 49 to 83 years] and 10 males [50 to 85 years]) undergoing hip arthroplasty for primary OA and from 10 patients (10 females [74 to 87 years]) undergoing hip arthroplasty for a fractured neck of femur (designated OP). For the control group, trabecular bone from the IT region was obtained during 21 autopsies (11 females [43 to 85 years] and 10 males [50 to 85 years]) of individuals who were known not to have suffered from any chronic condition or disease that may have affected the skeleton. In selecting the OA, OP and control individuals, those with a known history of medication that might have affected bone metabolism were excluded. Informed consent was obtained for the collection of these specimens, with approval from the Royal Adelaide Hospital Research Ethics Committee (protocol number 030309).

The surgical and autopsy femoral heads were graded for OA according to the criteria of Collins [40]. Primary OA femoral heads were either grade III or IV, and the graded autopsy femoral heads were not worse than grade II and predominantly were grade I. Surgical IT trabecular bone specimens from OA and OP individuals were collected within 12 to 24 hours (stored at 4°C in sterile RNase-free phosphate-buffered saline). Control bone was collected within 24 to 72 hours after death.

Trabecular bone in the IT region of the proximal femur, including the marrow, was sampled, permitting analysis of the total contribution of the bone microenvironment. The IT region was also chosen because the trabecular structure in this region depends on stresses in the proximal femoral shaft, while being unaffected by the secondary sclerotic and cystic changes that are often seen in the OA femoral head as the destruction of the cartilage proceeds. By comparing the OA and OP samples with control samples, the contribution to changes in gene expression associated with surgery as opposed to autopsy could be assessed.

### RNA extraction

For total RNA extraction, the trabecular bone samples were rinsed briefly in diethylpyrocarbonate-treated water and then separated into small fragments, containing bone and bone marrow, using bone cutters. Total RNA was extracted as described previously [35,41]. Briefly, bone fragments were placed in 4 mol/l guanidinium thiocyanate solution and homogenized using an Ultra-Turrax (TP 18–10; Janke & Kunkel, IKA-WERK, Staufen, Germany), and the mixture was clarified by centrifugation (1,000 × *g *for 5 min). After addition of 0.1 vol of 2 mol/l sodium acetate (pH 4.0), the mixture was vortexed and the RNA extracted with 1 vol of phenol and 0.2 vol of chloroform/isoamylalcohol (49:1). Total RNA was precipitated with isopropanol, resuspended in 1 × 10 mmol/l Tris-HCl/1 mmol/l EDTA containing 0.1 vol of 3 mol/l sodium acetate (pH 5.2) and then re-extracted with 0.5 vol phenol, followed by 0.5 vol chloroform/isoamylalcohol. The RNA was then precipitated with 3 vol of 4 mol/l sodium acetate (pH 7.0), to remove contaminating proteoglycans, at -20°C overnight. Total RNA was recovered by centrifugation, washed with 75% ethanol, air dried, dissolved in diethylpyrocarbonate-treated water, and stored at -80°C until further use. RNA concentration and purity (260/280 absorbance ratio) were determined by spectrophotometry. RNA integrity was confirmed by visualization on ethidium bromide stained 1% weight/vol agarose-formaldehyde gels.

### Microarray

RNA was further purified using RNeasy columns (Qiagen, Hilden, Germany), in accordance with the manufacturer's instructions. RNA (5 μg) was amplified using a Message Amp II kit (Ambion, Austin, TX, USA) with indirect, amino allyl mediated incorporation of either Cy3 or Cy5 dyes (Amersham Biosciences, Piscataway, NJ, USA), in accordance with the manufacturer's instructions. A Compugen Human 19K-oligo library (Jamesburg, NJ, USA) spotted onto Corning glass slides (Lowell, MA, USA) by the Adelaide Microarray facility (AMF) was used in this study. The Compugen human oligo library consisted of 17,260 oligonucleotide 65-mers each representing a single human gene. The slides were interrogated by competitive hybridization with 5 μg each of Cy3 and Cy5 labelled pairs of OA-control, OA-OP, or OP-control amplified RNA samples. The sample pairs used in the microarray analysis are listed in Table 1. Sample pairs were age-matched as closely as possible.

**Table 1:** Control versus OA versus OP sample microarray comparisons

		Sample pair					
		
Slide	GEO accession number	ID	Status	Age (years)	ID	Status	Age (years)
1	GSM207548	1	CTL	85	12	OA	83
2	GSM207549	2	CTL	83	13	OA	82
3	GSM207810	2	CTL	83	14	OA	82
4	GSM207811	3	CTL	72	15	OA	78
5	GSM207550	4	CTL	72	16	OA	77
6	GSM207812	5	CTL	68	21	OA	68
7	GSM207552	6	CTL	68	17	OA	66
8	GSM207553	7	CTL	60	18	OA	60
9	GSM207554	8	CTL	56	19	OA	56
10	GSM207555	9	CTL	43	20	OA	49
11	GSM208577	37	CTL	85	47	OA	85
12	GSM208575	38	CTL	73	48	OA	77
13	GSM208578	39	CTL	71	49	OA	73
14	GSM208576	40	CTL	71	50	OA	70
15	GSM208579	41	CTL	70	51	OA	69
16	GSM208583	42	CTL	69	52	OA	63
17	GSM208580	43	CTL	64	53	OA	63
18	GSM208582	44	CTL	60	54	OA	62
19	GSM208581	45	CTL	57	55	OA	57
20	GSM208584	46	CTL	50	56	OA	50
21	GSM207805	26	OP	91	22	OA	87
22	GSM207813	27	OP	87	12	OA	83
23	GSM207803	34	OP	87	13	OA	82
24	GSM207804	28	OP	84	23	OA	79
25	GSM207808	29	OP	81	24	OA	78
26	GSM207806	35	OP	78	15	OA	78
27	GSM207807	36	OP	78	16	OA	77
28	GSM207556	35	OP	78	25	OA	73
29	GSM208574	32	OP	74	21	OA	68
30	GSM207809	33	OP	74	17	OA	66
32	GSM207798	2	CTL	83	27	OP	87
33	GSM207557	2	CTL	83	34	OP	87
34	GSM207796	10	CTL	83	28	OP	84
35	GSM207797	11	CTL	74	29	OP	81
36	GSM207799	4	CTL	72	36	OP	78
37	GSM207800	3	CTL	72	30	OP	77
38	GSM207551	3	CTL	72	31	OP	75
39	GSM207801	5	CTL	68	32	OP	74
40	GSM207802	6	CTL	68	33	OP	74

'Slide' indicates the microarray slide comparison. Slides 1 to 10 are control (CTL)-osteoarthritis (OA) female sample pairs, slides 11 to 20 are CTL-OA male sample pairs, slides 21 to 30 are OA-osteoporosis (OP) female sample pairs and slides 32 to 40 are CTL-OP female sample pairs. GEO, Gene Expression Omnibus; ID, individual/sample.

A biological dye-swap strategy was employed rather than a replicate dye swap strategy. This involved swapping of Cy3 and Cy5 labelling of the samples in each pair for each group of paired samples to balance for potential dye incorporation and signal intensity bias. It also reduced the number of slides required for the experiment and maximized the statistical power of the experiment with regard to analyzing the biological differences between samples.

Hybridization and washing of slides was carried out according to methods described on the AMF website [42]. The microarray slides were scanned twice at slightly different PMT voltage using a GenePix 4000B Scanner driven by GenePix Pro 4.0 (Axon Instruments, Foster City, CA, USA). All analyses were performed using the statistical programming and graphics environment R [43]. The 'SPOT' software package [44] was used to identify spots using the adaptive segmentation method and subtract backgrounds utilizing the morphological opening approach [45,46]. Data analysis was performed in R using Bioconductor [47]. The Loess print tip method was used to correct for dye bias and intensity within each group of adjacent spots printed by one pin [48]. Linear modelling was performed using the linear models for microarray analysis (LIMMA) package of Bioconductor [49]. Differentially expressed genes were ranked on moderated t statistics, and those with t scores above 3 were followed up further. The moderated t-statistic score is based on the ratio of the log_2 _fold change to its standard error. Because there is no consensus on appropriate adjustment of *P *values in the context of microarrays, genes of interest were chosen based on a combination of statistical and biological indicators. Microarray data have been deposited in the Gene Expression Omnibus [50] and are accessible through Gene Expression Omnibus series number GSE8406.

### Real-time PCR

First-strand reverse transcription cDNA synthesis was performed on 1 μg amplified RNA from each sample using a first-strand cDNA synthesis kit with Superscript II (Invitrogen, Carlsbad, CA, USA) and 250 ng random hexamer primer (Geneworks, Adelaide, SA, Australia), in accordance with the manufacturer's instructions. Template cDNA (1 μl of 1/100 dilution of cDNA) was amplified using iQ SYBR Green Supermix (BioRad, Hercules, CA, USA) on a Rotor-Gene thermocycler (Corbett Research, Mortlake, NSW, Australia). The reactions were incubated at 94°C for 10 min for 1 cycle, and then 94°C (20 seconds), 60°C, or 65°C (*ADAMTS4 *and *MMP25 *only; 20 seconds) and 72°C (30 seconds) for 40 cycles. This set of cycles was followed by an additional extension step at 72°C for 5 minutes. All PCR reactions were validated by the presence of a single peak in the melt curve analysis, and amplification of a single specific product was further confirmed by electrophoresis on a 2.5% weight/vol agarose gel. Primers were designed for each gene that primed in separate exons and spanned at least one intron to avoid contaminating amplification from genomic DNA. Primers were obtained from Geneworks. Amplicons were designed to be in the 100 to 200 base pairs size range. GenBank accession numbers for gene sequences and primer sequences are provided in Table 2. Real-time PCR validation was carried out using the 2^-ΔΔCT ^method [51]. Reactions were performed in duplicate. Normalized gene expression values for each gene based on cycle threshold (C_T_) values for each of the genes and the housekeeping gene *GAPDH *were generated. Mean ± standard deviation (SD) values were generated from eight samples from each group of either OA or control samples tested.

**Table 2:** GenBank accession numbers and primer sequences

Gene/primer (GenBank accession number)	Forward	Reverse
*GAPDH *(NM_002046)	ACCCAGAAGACTGTGGATGG	CAGTGAGCTTCCCGTTCAG
*ADAMTS4 *(NM_005099)	GGCTACTACTATGTGCTGGAGC	TCCGCACACCATGCACTTGTCA
*ADM *(NM_001124)	GGATGAAGCTGGTTTCCGTC	GACTCAGAGCCCACTTATTC
*ADFP *(NM_001122)	GTTGCCAATACCTATGCCTG	CAGTAGTCGTCACAGCATCT
*CD14 *(NM_000591)	GAGGTTCGGAAGACTTATCG	ATCTTCATCGTCCAGCTCAC
*COL4A1 *(NM_001845)	TAGAGAGGAGCGAGATGTTC	GTGACATTAGCTGAGTCAGG
*CTNNB1 *(NM_001904)	GGTGCTATCTGTCTGCTCTAGT	GACGTTGACTTGGATCTGTCAGG
*FST *(NM_006350)	GGCAAGATGTAAAGAGCAGC	CATTATTGGTCTGGTCCACC
*GADD45B *(NM_015675)	TTGCAACATGACGCTGGAAG	CATTCATCAACTTGGCCGAC
*IBSP *(NM_004967)	CAATCCAGCTTCCCAAGAAG	CTTCTGCTTCGCTTTCTTCG
*INHBA *(NM_002192)	GAACTTATGGAGCAGACCTC	TGCCTTCCTTGGAAATCTCG
*INSIG1 *(NM_005542)	TGTATCGACAGTCACCTCGGA	GGACAGCTGGACATTATTGGC
*ITGB2 *(NM_000211))	AAGTGACGCTTTACCTGCGA	CCTGAGGTCATCAAGCATGG
*KLF6 *(NM_001300)	TGTGCAGCATCTTCCAGGAG	AACGTTCCAGCTCTAGGCAG
*MEPE *(NM_020203)	GCAAAGCTGTGTGGAAGAGCAGA	CCCTTATTCTCACTGGCTTCAG
*MMP25 *(NM_004142)	ATGTCACCGTCAGCAACGCA	CGGTCTTGATGCTGTTCTTG
*MT2A *(NM_005953)	GCAAATGCACCTCCTGCAAG	GTGGAAGTCGCGTTCTTTAC
*NHERF1 *(NM_004252)	TCACCAATGGGGAGATACAG	GTCTTGGGAATTCAGCTCCT
*PTEN *(NM_000314)	AAGACAAAGCCAACCGATAC	GAAGTTGAACTGCTAGCCTC
*RUNX2 *(NM_004348)	TGATGACACTGCCACCTCTG	GGGATGAAATGCTTGGGAAC
*S100A4 *(NM_002961)	GTCAGAACTAAAGGAGCTGC	TGTTGCTGTCCAAGTTGCTC
*SMAD3 *(NM_005902)	TTCAACAACCAGGAGTTCGC	TACTGGTCACAGTA
*STC1 *(NM_003155)	CCTGTGACACAGATGGGATG	GAATGGCGAGGAAGACCTTG
*TIMP4 *(NM_003256)	TTGACTGGTCAGGTCCTCAGT	GGTACTGTGTAGCAGGTGGT
*TWIST1 *(NM_000474)	TCAGCAGGGCCGGAGACCTAGAT	GTCTGGGAATCACTGTCCAC
*WNT5B *(AY009399)	ACCCTACTCTGGAAACTGTC	TAAACATCTCGGGTCTCTGC

'Slide' indicates the microarray slide comparison. Slides 1 to 10 are control (CTL)-osteoarthritis (OA) female sample pairs, slides 11 to 20 are CTL-OA male sample pairs, slides 21 to 30 are OA-osteoporosis (OP) female sample pairs and slides 32 to 40 are CTL-OP female sample pairs. GEO, Gene Expression Omnibus; ID, individual/sample.

### Statistical analysis

The statistical significance of the differences between the means of the OA and control or OP gene expression values was determined using Student's *t*-test. The critical value for significance was chosen as *P *< 0.05.

## Results

### Microarray analysis of OA, control and OP bone samples

**Figure 1:**
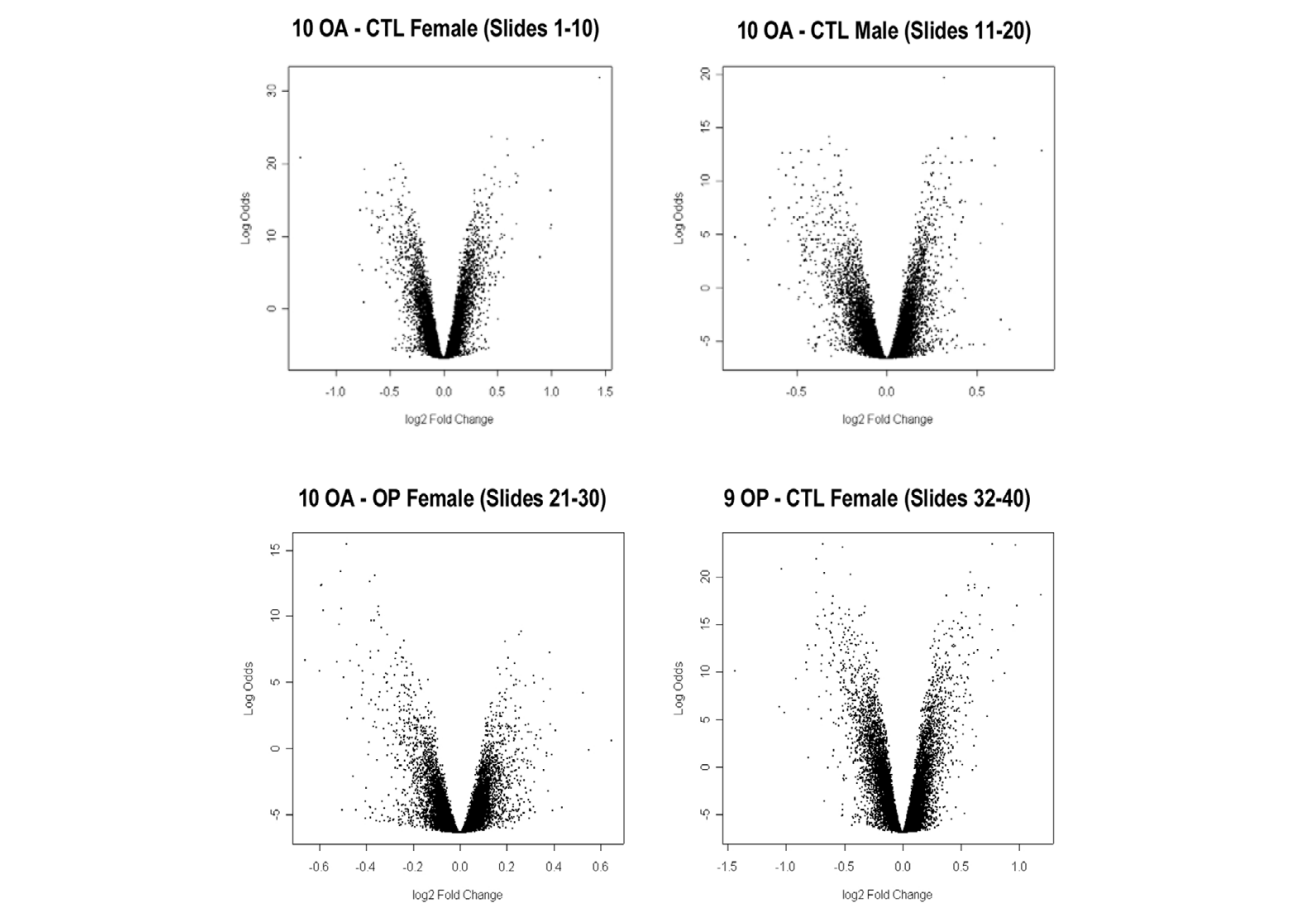
Bayesian statistical analysis of differentially expressed genes using LIMMA. Log odds (LOD) score versus log_2 _fold change volcano plots of differentially expressed genes from each of the four groups of sample pair comparisons. CTL, control; LIMMA, linear models for microarray analysis; OA, osteoarthritis; OP, osteoporosis.

This study used Compugen human 19K-oligo human microarray slides to compare the gene expression profiles of OA, control and OP bone samples, with the aim being to identify altered gene expression in OA bone. Microarray analysis was conducted in four sets of samples (39 comparisons in total), comprising 10 OA-control female sample pairs, 10 OA-control male sample pairs, 10 OA-OP female sample pairs and 9 OP-control female sample pairs. Samples from individuals with a range of ages were analyzed in each group, but with sample pairs age-matched as closely as possible (Table 1). Bayesian statistical analysis was carried out using LIMMA to identify statistically significant differentially expressed genes between OA, control and OP bone. Log odds score versus log_2 _fold change volcano plots of differentially expressed genes from each of the four groups of sample pair comparisons are shown in Figure 1. The log odds (or B statistic) score is the log odds that that gene is differentially expressed. The log_2 _fold change represents the fold change in expression of the gene. Small levels of differential expression (ranging from 0.38-fold to 2.83-fold change in expression) were detected, with several hundred differentially expressed genes present in each grouping, with t scores above 6. The moderated t-statistic score is based on the ratio of the log_2 _fold change to its standard error.

### Identification and functional classification of top-ranking differentially expressed genes in OA bone

By comparing the lists of ranked differentially expressed genes from each of the four initial groupings, we were able to identify a group of differentially expressed genes that was more likely to be associated with the OA disease process. This group of genes was assembled by filtering out genes that were similarly regulated between OA-control and OP-control samples in order to remove genes that were more likely to be differentially expressed because of potential differences caused by sourcing bone at surgery versus autopsy. Because there were also very few significant differences in gene expression between the male and female OA-control groups, these data were combined because it strengthened the statistical significance of the genes identified as differentially expressed. Using these selection processes, several hundred genes from each initial grouping was reduced to a list of 150 differentially expressed genes in OA bone with t scores above 4.

Gene function and pathway analyses were carried out by searching the National Centre for Biotechnology Information database [52] and by using various analysis programs including OntoExpress [53] and Gostat [54]. We were able to identify a group of 62 top-ranking OA differentially expressed genes from within the initial list of 150 genes, which have known or suspected roles (direct or indirect via angiogenesis) in influencing bone development or bone remodelling (Table 3). For many of the genes both osteogenic and angiogenic roles have been described. In addition, a subset of these genes, particularly those that encode secreted, cell surface and extracellular matrix molecules, also have potential chondrogenic functions, consistent with the proposal that an altered OA subchondral bone microenvironment could interfere with cartilage metabolism.

**Table 3:** Differentially expressed genes in OA bone with roles in osteogenesis, angiogenesis and chondrogenesis

									Real-time PCR	
Rank	GenBank	Role	Cell type	Pathway	Symbol	Name	t OA/CTL	t OA/OP	Symbol	OA/CTL
									*ADAMTS4*	0.11
2	NM_001124	A, B, C	OB, OC, OS, CB	TGF-β/BMP	*ADM*	Adrenomedullin	-12.620	-7.904	*ADM*	0.09
3	NM_002450	B	OB		*MT1L*	Metallothionein 1L	-8.342	-6.843		
4	NM_016109	A		WNT	*ANGPTL4*	Angiopoietin-like 4	-6.165	-8.052		
5	NM_015675	B	OB, OC, CB	TGF-β/BMP	*GADD45B*	Growth arrest and DNA-damage-inducible, beta	-8.102	-5.985	*GADD45B*	0.15
7	NM_000358	A, B		TGF-β/BMP	*TGF-BI*	Transforming growth factor, beta-induced	-6.915	-5.741		
9	NM_017817				*RAB20*	RAB20, member RAS oncogene family	-5.147	-6.982		
10	NM_004967	B, C	OB, CB	WNT & TGF-β/BMP	*IBSP*	Integrin-binding sialoprotein	-5.088	-5.865	*IBSP*	0.25
12	AB014526	B	OB		*MFAP3L*	Microfibrillar-associated protein 3-like	5.284	4.983		
13	NM_004142	A, B, C	OB, OC, CB	WNT	*MMP25*	Matrix metalloproteinase 25	4.942	7.183	*MMP25*	2.60
15	NM_002025	B		TGF-β/BMP	*FMR2*	Fragile × mental retardation 2	4.915	4.851		
18	NM_005953	B	OB		*MT2A*	Metallothionein 2A	-4.638	-7.071	*MT2A*	0.18
20	NM_013258	A, B	M OC		*PYCARD*	PYD and CARD domain containing	6.567	4.355		
21	NM_014467				*SRPX2*	Sushi-repeat-containing protein, X-linked 2	-4.333	-4.918		
22	NM_003155	A, B, C	OB, OC, CB		*STC1*	Stanniocalcin 1	-6.656	-4.274	*STC1*	0.16
23	NM_001109	B	OC		*ADAM8*	A disintegrin and metalloproteinase domain 8	5.426	4.269		
24	NM_020203	B	OB, OS	TGF-β/BMP	*MEPE*	Matrix, extracellular phosphoglycoprotein with ASARM motif	-5.036	-4.263	*MEPE*	0.11
28	S68954	B	OB		*MT1G*	Metallothionein 1G	-4.202	-7.630		
29	AK026438	B	OB		*GALNT4*	Polypeptide N-acetylgalactosaminyltransferase 4	5.259	4.195		
30	NM_000474	B	OB	WNT	*TWIST1*	Twist homolog 1	-4.194	-4.910	*TWIST1*	0.31
33	NM_000655	B	M		*SELL*	Selectin L	4.852	4.179		
36	NM_002341	A, B, C	OB, CB		*LTB*	Lymphotoxin beta	4.465	4.086		
37	NM_003226	A			*TFF3*	Trefoil factor 3	4.080	4.074		
42	NM_000607	B	M		*ORM1*	Orosomucoid 1	4.284	3.982		
46	NM_005542				*INSIG1*	Insulin induced gene 1	-7.756	-3.878	*INSIG1*	0.55
49	NM_001302	B	M		*CORT*	Cortistatin	-6.713	-3.843		
52	NM_000598	A, B, C	OB, OC, CB	WNT & TGF-β/BMP	*IGFBP3*	Insulin-like growth factor binding protein 3	-3.772	-3.893		
54	NM_012479	A, B	OB		*YWHAG*	Tyrosine 3-/tryptophan 5-monooxygenase activation protein, gamma	-3.759	-5.355		
59	NM_014624	B	OB	WNT	*S100A6*	S100 calcium binding protein A6	8.214	3.710		
61	NM_006732	A, B	OB		*FOSB*	FBJ murine osteosarcoma viral oncogene homolog B	3.682	3.926		
64	NM_002961	A, B	OB	WNT	*S100A4*	S100 calcium binding protein A4	4.407	3.596	*S100A4*	2.43
65	NM_016184	B	M		*CLECSF6*	C-type lectin domain family 4, member A	4.575	3.594		
68	NM_006184	B			*NUCB1*	Nucleobindin 1	-3.551	-5.513		
69	NM_001300	A		WNT & TGF-β/BMP	*KLF6*	Kruppel-like factor 6	-4.047	-3.544	*KLF6*	0.41
70	NM_004864	A, B	OB, CB	TGF-β/BMP	*GDF15*	Growth differentiation factor 15	-3.541	-4.269		
73	U79271	B	OB, OC	WNT	*AKT3*	V-akt murine thymoma viral oncogene homolog 3	3.507	4.876		
76	S83282	A, B	OB, OC		*MIF*	Macrophage migration inhibitory factor	-3.654	-3.496		
81	NM_005213	B	OC		*CSTA*	Cystatin A	4.450	3.447		
82	NM_000647	A, B	M OC		*CCR2*	Chemokine (C-C motif) receptor 2	7.277	3.446		
85	NM_001911	A			*CTSG*	Cathepsin G	8.474	3.411		
87	NM_001122			WNT	*ADFP*	Adipose differentiation-related protein	-3.368	-10.219	*ADFP*	0.21
92	NM_006254	A, B	OB		*PRKCD*	Protein kinase C, delta	5.549	3.319		
94	NM_002162	A			*ICAM3*	Intercellular adhesion molecule 3	3.304	5.798		
95	NM_001885	B	OB, OC		*CRYAB*	Crystallin, alpha B	-3.296	-7.616		
96	D17152	A		WNT	*SOD2*	superoxide dismutase 2	-6.347	-3.278		
102	NM_003256	A		WNT	*TIMP4*	Tissue inhibitor of metalloproteinase 4	-6.319	-3.250	*TIMP4*	0.08
105	NM_000698		M		*ALOX5*	Arachidonate 5-lipoxygenase	3.229	4.736		
106	NM_014029	B	OC		*RAC2*	Rho family, small GTP binding protein Rac2	3.227	5.325		
108	NM_003670	B, C	OB, CB	TGF-β/BMP	*DEC1*	Differentially expressed in chondrocytes 1	-3.203	-3.783		
109	NM_002067	B	OB		*GNA11*	Guanine nucleotide binding protein, alpha 11	-4.353	-3.202		
115	AF263545	B	OB		*SLC14A1*	Solute carrier family 14, member 1	3.163	5.759		
120	NM_005127	B	M		*CLECSF2*	C-type lectin domain family 2, member B	-7.330	-3.141		
123	NM_004252	B		WNT	*NHERF1*	Sodium/Hydrogen exchanger regulatory factor 1	5.280	3.138	*NHERF1*	1.71
124	NM_006186	B	OB		*NR4A2*	Nuclear receptor subfamily 4, group A, member 2	-3.128	-3.354		
133	NM_000104	B	OC		*CYP1B1*	Cytochrome P450, family 1, subfamily B, 1	-3.075	-5.148		
136	NM_003005	B			*SELP*	Selectin P	8.740	3.066		
139	NM_004334	B	OC		*BST1*	Bone marrow stromal cell antigen 1 (CD157)	3.037	3.601		
141	AK023619	A			*CRIM1*	Cysteine-rich motor neuron 1	-3.043	-3.271		
143	NM_013332	A		WNT	*HIG2*	Hypoxia-inducible protein 2	-6.019	-3.035		
144	NM_001845	A, B		TGF-β/BMP	*COL4A1*	Collagen, type IV, alpha 1	-3.028	-8.960	*COL4A1*	0.57
146	NM_016224	A, B	OB	WNT	*SNX9*	Sorting nexin 9	-3.025	-6.086		

Rank' indicates the ranking within the top 150 differentially expressed genes in osteoarthritis (OA) bone compared with control (CTL) and osteoporosis (OP) bone. 'Role' indicates the known or suspected role of the gene: A, angiogenic; B, osteogenic; and C, chondrogenic. 'Cell type' indicates the cell type that the gene is expressed in or affects: OB, osteoblast, OC, osteoclast, OS, osteocyte, CB, chondroblast, or M, monocyte. 't OA/CTL' is the t score of OA compared with CTL differential expression of gene: a positive value indicates upregulation in OA and a negative one indicates downregulation in OA. 't OA/OP' is the t score of OA compared with OP differential expression of gene: a positive value indicates upregulation in OA and a negative one indicates downregulation in OA. The moderated t-statistic score is based on the ratio of the log_2 _fold change to its standard error. 'OA/CTL' under 'Real-time PCR' indicates the fold change in gene expression expressed as ratio of OA to CTL.

Although many of the genes identified in this analysis have pleiotropic effects in bone and other tissues, it was of interest that many of the top-ranking differentially expressed genes in OA bone have known or suspected roles in osteoblast and osteocyte differentiation and function. These genes included *ADAMTS4*, *ADM*, *GADD45B*, *IBSP*, *MMP25*, *MT2A*, *STC1*, *MEPE*, *TWIST1*, *IGFBP3*, *S100A4*, *AKT3 *and *COL4A1*. There was also a group of differentially expressed genes in OA bone that have known or potential roles in osteoclast function, such as the previously mentioned osteoblast-related genes *ADAMTS4*, *GADD45B*, *STC1 *and *IGFB3*, as well as *ADAM8*, *CCR2*, *CSTA*, *RAC2*, *CRYAB *and *CYP1B*. Functionally, within the list of genes given in Table 3, there are genes encoding secreted molecules (*ADM*, *ANGPTL4*, *STC1*, *CORT*, *IGFBP3 *and *MIF*), cell surface molecules (*SELL*, *ICAM3*, *SELP*, *CRIM1*, *CLECSF6*, *CLECSF2*, *CCR2 *and *SLC14A1*), intracellular signalling molecules (*RAB20*, *YWHAG*, *RAC2*, *NHERF1*, *GNA11 *and *SNX9*), protein kinases (*AKT3 *and *PRKCD*), calcium and metal ion binding proteins (*S100A4*, *S100A6, MT1L*, *MT2A *and *MT1G*), transcription factors (*TWIST1*, *FMR2*, *KLF6*, *NR4A2 *and *DEC1*), and both enzymatic (*ADAMTS4*, *MMP25*, *ADAM8*, *TIMP4*, *GALNT4 *and *CTSG*) and structural (*TGFBI*, *IBSP*, *MEPE*, *MFAP3L *and *COL4A1*) extracellular matrix molecules.

Because of the small absolute differences in gene expression between the bone tissue samples, real-time PCR was used to confirm a selection of the differentially expressed genes identified by the microarray analysis of OA, control and OP bone. The real-time PCR results (depicted as fold differential expression) are shown alongside the microarray results in Table 3. In total, the differential expression levels of 20 genes were examined using real-time PCR. Results for 16 genes reached statistical significance (*P *< 0.01) for differential expression between OA and control bone. The differential expression of four genes (*TGFBI*, *S100A6*, *SLC14A1 *and *SNX9*) could not be confirmed. The female control samples 1–8 (age range 56 to 85 years, mean [ ± SD] age 70.5 ± 10 years) and female OA samples 12–19 (age range 56 to 83 years; mean age 73 ± 10.8 years) were used to confirm the microarray data by real-time PCR (Table 1). The mean age of the OA group did not differ significantly from that in the control group. Interestingly, although the microarray expression ratios were quite small (ranging from 0.62-fold change to 1.47-fold change in expression), the fold difference in expression identified using the real-time PCR reactions was significantly greater in most cases (ranging from 0.08-fold change to 2.6-fold change in expression). This probably reflects differences in sensitivity between the two techniques [55,56]. The difference is probably also accentuated by the competitive pair-wise comparison of samples used by the microarray platform in this study compared with the individual gene/*GAPDH *C_T _expression ratio values generated using real-time PCR. Encouragingly, there was a high confirmation rate with the real-time PCR and consistency between the microarray and PCR detection of expression ratio differences for each of the genes analyzed, suggesting that the majority of the genes identified by the microarray are *bona fide *differentially expressed genes in OA bone.

### Altered expression of WNT and TGF-β/BMP signalling pathway component and target genes in OA bone

A significant number of the top-ranking differentially expressed genes in OA bone were identified as WNT signalling pathway targets (Table 3). WNT targets included upregulated genes such as *MMP25 *and *S100A4*, and downregulated genes such as *IBSP*, *TWIST1 *and *TIMP4*. The altered expression of these genes suggests that WNT signalling may be perturbed in the OA bone microenvironment. This was apparently borne out by closer examination of the extended list of differentially expressed genes in OA bone, which revealed further WNT signalling pathway components and modulators such as *WNT5B*, *FZD3*, *SFRP5*, *APC*, *AXIN2*, *PTEN *and *NHERF1*. These genes, and additional WNT target genes such as *CD14*, *APOE*, *ID1*, *IL6*, *FST *and *RUNX2*, are listed in Table 4. The differences in expression of this group of genes (t scores above 3) in general were not as pronounced as that seen for the target genes identified from within the top-ranking 150 genes.

**Table 4:** WNT and TGF-β/BMP signalling pathway components and target genes differentially expressed in OA bone

							Real-time PCR	
							
GenBank	Role	Cell type	Symbol	Name	t OA/CTL	t OA/OP	Symbol	OA/CTL
WNT pathway components and modulators								
AY009399	B, C	OB, OC, CB	*WNT5B*	Wingless-type MMTV integration site family, member 5B	3.719	0.717	*WNT5B*	2.52
NM_002332	A, B, C	OB, OC, CB	*LRP1*	Low density lipoprotein-related protein 1	3.062	-7.071		
NM_002333			*LRP3*	Low density lipoprotein-related protein 3	5.950	1.146		
NM_017412			*FZD3*	Frizzled homolog 3	-3.677	0.835		
AF086500			*FZD8*	Frizzled homolog 8	3.024	-1.557		
NM_003015			*SFRP5*	Secreted frizzled-related protein 5	-4.338	1.154		
NM_004655			*AXIN2*	Axin 2	-3.123	2.490		
NM_000038			*APC*	Adenomatosis polyposis coli	-5.171	1.155		
AK023892			*DAAM1*	Dishevelled associated activator of morphogenesis 1	-6.704	2.850		
NM_000314	A, B	OB, OC	*PTEN*	Phosphatase and tensin homolog	-5.288	-0.216	*PTEN*	0.30
AF028823			*TIP1*	Tax interaction protein 1	5.057	-5.674		
AB006630			*TCF20*	Transcription factor 20	8.559	2.719		
AK026898	B	M	*FOXP1*	Forkhead box P1	-5.122	1.761		
WNT inducible/target genes								
NM_000591	B	OB, OC, M	*CD14*	CD14 antigen	5.965	-7.036	*CD14*	3.47
NM_004039	A, B	OB, OC, M	*ANXA2*	Annexin A2	5.716	-7.601		
NM_000211	B	OC	*ITGB2*	Integrin, beta 2 (CD18)	6.104	-1.243	*ITGB2*	2.95
NM_018456			*EAF2*	ELL associated factor 2	6.450	0.884		
NM_000041	B	OB, M	*APOE*	Apolipoprotein E	6.896	-2.391		
AB012643	B	OB	*ALPL*	Alkaline phosphatase, liver/bone/kidney	-5.942	-1.213		
NM_002229	B	OB	*JUNB*	Jun B proto-oncogene	5.053	-3.804		
NM_003377	A, B		*VEGFB*	Vascular endothelial growth factor B	3.238	-3.772		
NM_005429	A, B		*VEGFC*	Vascular endothelial growth factor C	5.304	0.473		
NM_000963	B	OB	*PTGS2*	Prostaglandin-endoperoxide synthase 2	4.346	-0.842		
NM_005194	B	OB	*CEBPB*	CCAAT/enhancer binding protein, beta	4.273	-4.092		
AL353944	A, B	OB	*ID1*	Inhibitor of DNA binding 1	3.923	-1.996		
NM_000900	B	OB	*MGP*	Matrix Gla protein	2.791	-3.073		
M14584	A, B, C	OB, OC, CB	*IL6*	Interleukin 6	2.589	-3.612		
NM_006350	B, C	OB, CB	*FST*	Follistatin	-2.137	-7.912	FST	0.38
AL353944	B	OB	*RUNX2*	Runt-related transcription factor 2	1.611	1.050	RUNX2	2.07
TGF-β/BMP pathway components and modulators								
M38449	A, B, C	OB, OC, CB	*TGFB1*	Transforming growth factor, beta 1	2.739	3.054		
AK021486	B, C	OB, CB	*BMP5*	Bone morphogenetic protein 5	-10.314	-2.263		
NM_002192	A, B, C	OB, OC, CB	*INHBA*	Inhibin, beta A	-7.699	-0.389	*INHBA*	0.31
NM_006350	B, C	OB, CB	*FST*	Follistatin	-2.137	-7.912	*FST*	0.38
NM_004612	B		*TGFBR1*	Transforming growth factor, beta receptor I	-4.915	1.049		
NM_000118	A		*ENG*	Endoglin	4.131	-2.461		
NM_001105	B	OB	*ACVR1*	Activin A receptor, type I	3.791	-0.941		
NM_003573			*LTBP4*	Latent transforming growth factor beta binding protein 4	-4.060	2.623		
NM_005902	A, B	OB	*SMAD3*	SMAD, mothers against DPP homolog 3	4.119	-3.582	*SMAD3*	2.64
NM_005359	A, B	OB	*SMAD4*	SMAD, mothers against DPP homolog 4	3.957	0.156		
NM_002165	A, B	OB	*ID1*	Inhibitor of DNA binding 1	3.923	-1.996		
NM_002229	B	OB	*JUNB*	Jun B proto-oncogene	5.053	-3.804		
NM_005655	B	OB	*KLF10*	Kruppel-like factor 10	4.443	-4.449		
NM_006037	B	OB	*HDAC4*	Histone deacetylase 4	8.475	2.468		
NM_000168	B	OB	*GLI3*	GLI-Kruppel family member GLI3	-4.675	-0.298		
AL353944	B	OB	*RUNX2*	Runt-related transcription factor 2	1.611	1.050	*RUNX2*	2.07
TGF-β/BMP inducible/target genes								
AK001052	B	OB	FGFR1	Fibroblast growth factor receptor 1	4.234	1.993		
NM_001553	A		IGFBP7	Insulin-like growth factor binding protein 7	5.137	-6.302		
NM_000211	B	OC	ITGB2	Integrin, beta 2 (CD18)	6.104	-1.243	ITGB2	2.95
AK001060	B, C	OB, CB	DCN	Decorin	-6.325	2.628		
NM_000177	B	OC	GSN	Gelsolin	4.725	-2.387		
X55525	B	OB	COL1A2	Collagen, type I, alpha 2	5.538	-3.633		
NM_003118	A, B	OB	SPARC	Secreted protein, acidic, cysteine-rich	3.188	-1.844		
NM_004407	B	OB, OS	DMP1	Dentin matrix acidic phosphoprotein	-3.523	-1.397		
NM_001831	A, B		CLU	Clusterin	4.803	-2.030		
Not represented on Compugen human 19K microarray								
NM_001904	A, B	OB, OC	*CTNNB1*	Catenin beta 1			*CTNNB1*	2.38

'Role' indicates the known or suspected role of the gene: A, angiogenic; B, osteogenic; and C, chondrogenic. 'Cell type' indicates the cell type that the gene is expressed in or affects: OB, osteoblast, OC, osteoclast, OS, osteocyte, CB, chondroblast, or M, monocyte. 't OA/CTL' is the t score of osteoarthritis (OA) compared with control (CTL) differential expression of gene: a positive value indicates upregulation in OA and a negative one indicates downregulation in OA. 't OA/OP' is the t score of OA compared with osteoporosis (OP) differential expression of gene: a positive value indicates upregulation in OA and a negative one indicates downregulation in OA. The moderated t-statistic score is based on the ratio of the log_2 _fold change to its standard error. 'OA/CTL' under 'Real-time PCR' indicates the fold change in gene expression expressed as ratio of OA to CTL. BMP, bone morphogenic protein; TGF, transforming growth factor.

In addition, a significant number of TGF-β/BMP signalling pathway target genes were identified as being differentially expressed in OA bone (Table 3). TGF-β/BMP signalling pathway targets included downregulated genes such as *ADAMTS4*, *ADM*, *GADD45B*, *MEPE *and *COL4A1*. The altered expression of these genes also suggests that TGF-β/BMP signalling may be perturbed in the OA bone microenvironment. Additional evidence for this was that genes for TGF-β/BMP signalling pathway components and modulators, such as *TGFB1*, *BMP5*, *INHBA*, *SMAD3 *and *FST*, were also identified in the extended list of differentially expressed genes in OA bone. These genes, and additional TGF-β/BMP target genes identified, such as *COL1A2*, *GSN*, *DMP1 *and *ITGB2*, are listed in Table 4. The differences in expression of this group of genes (t scores above 3) was not as pronounced as the target genes identified from within the top-ranking 150 genes.

Like the top-ranking list of 150 differentially expressed genes in OA bone, many of the WNT and TGF-β/BMP signalling pathway related genes identified in Table 4 also have known or suspected roles in either osteoblast (*WNT5B*, *PTEN*, *CD14*, *SMAD3*, *RUNX2*, *ID1*, *HDAC4*, *TGFB1*, *BMP5, INHBA*, *DMP1 *and *FST*) or osteoclast (*CD14*, *PTEN*, *FOXP1*, *ANXA2*, *ITGB2*, *IL6 *and *GSN*) differentiation and function.

The differential expression of a selection of these WNT and TGF-β/BMP signalling pathway component and target genes was confirmed by real-time PCR. In total, the differential expression of 11 genes was examined by real-time PCR (Table 4). The differential expression of two genes (*LRP1 *and *IGFBP7*) could not be confirmed. However, results for the other nine genes reached statistical significance (*P *< 0.01) for differential expression between OA and control bone. *CTNNB1 *was assayed directly by real-time PCR because it was not represented on the Compugen H19K library. Seven of the remaining nine genes (with *FST *and *RUNX2 *being the exceptions) were represented in the top-ranking 300 genes differentially regulated in OA bone. These genes were tested, like those listed in Table 3, using female control samples 1 to 8 and female OA samples 12 to 19 (Table 1). The range of fold difference in expression identified by the real-time PCR reactions was slightly smaller (ranging from 0.3-fold change to 3.47-fold change in expression) than for the group of genes tested from the top ranking 150 genes in Table 3.

### Identification of differentially expressed OA genes between females and males

There is a higher incidence of primary hip OA in females than in males [1], and we were interested in identifying differences in gene expression between females and males that may contribute to this disparity. Therefore, we tested for differences between the OA-control female and male microarray datasets. Genes with the greatest difference in expression between females and males in the OA-control microarray comparisons are listed in Table 5. There were very few significant differences in gene expression between females and males. However, approximately 50 genes with t scores above 3, which included the top-ranking 20 genes with t scores above 4, were identified as being differentially expressed between females and males. Interestingly, a significant proportion of these genes have known or suspected roles in osteoclast-lineage cells and osteoclasts (*GSN*, *PTK9*, *VCAM1*, *ITGB2*, *GRN*, *ANXA2*, *PDE4A *and *FOXP1*). There are also genes with known roles in osteoblasts (*LTF*, *DF*, *PRKCG *and *TGFB1*). A number of the highest ranking differentially expressed genes between females and males in OA bone also involve WNT signalling pathway components, including *WNT5B*, along with the *EAF2 *and *CTBP2 *genes, which encode transcription factors that are involved in mediating WNT signalling.

**Table 5:** Genes differentially expressed between female and male OA and control bone samples

GenBank	Role	Cell type	Symbol	Name	t OAF/OAM	t OA/CTL	t OA/OP
Differentially expressed genes in top 20 regulated genes							
NM_000177	B	OC	*GSN*	Gelsolin	5.983	4.725	-2.387
NM_001078	B	OB, OC	*VCAM1*	Vascular cell adhesion molecule 1	5.158	-1.358	-2.377
NM_006764			*IFRD2*	Interferon-related developmental regulator 2	-5.081	4.274	2.162
NM_018456			*EAF2*	ELL associated factor 2	4.574	6.450	0.884
NM_002087	B	OC	*GRN*	Granulin	4.562	11.161	-2.008
NM_000211	B	OC	*ITGB2*	Integrin, beta 2 (CD18)	4.378	6.104	-1.243
NM_004203			*PKMYT1*	Protein kinase, membrane associated tyrosine/threonine 1	-4.373	-3.375	1.741
NM_001329			*CTBP2*	C-terminal binding protein 2	-4.339	1.548	4.057
NM_006202	B	OB, OC	*PDE4A*	Phosphodiesterase 4A	-4.131	-1.565	3.337
NM_017845			*COMMD8*	COMM domain containing 8	4.118	3.264	-0.398
AY009399	B, C	OB, OC, CB	*WNT5B*	Wingless-type MMTV integration site family, member 5B	4.107	3.719	0.717
NM_002822	B	OC	*PTK9*	PTK9 protein tyrosine kinase 9	4.099	-0.597	-5.894
NM_002343	B	OB	*LTF*	Lactotransferrin	4.096	4.918	1.882
Additional differentially expressed genes in top 50 regulated genes							
NM_005606	A, B	OB, OC	*LGMN*	Legumain	3.853	9.631	-4.470
NM_015946	A, B, C	OB, CB	*ITGA1*	Integrin, alpha 1	3.813	3.904	0.131
NM_004039	B	OB, OC	*ANXA2*	Annexin A2	3.754	5.716	-7.601
M14087	A, B	OB	*LGALS1*	Lectin, galactoside-binding, soluble,	3.705	4.583	-0.045
NM_006079	A, B	OB	*CITED2*	Cbp/p300-interacting transactivator with Glu/Asp-rich domain 2	3.591	8.580	-1.403
M38449	A, B, C	OB, OC, CB	*TGFB1*	Transforming growth factor, beta 1	-3.575	2.739	3.054
Z15114	A, B	OB	*PRKCG*	Protein kinase C, gamma	-3.570	0.018	3.741
NM_003639	B	OC	*IKBKG*	I kappa B kinase gamma	3.376	6.775	-1.704
AK026898	B	OC	*FOXP1*	Forkhead box P1	-3.348	-5.122	1.761
NM_001928	B	OB	*DF*	D component of complement (adipsin)	3.306	6.851	-0.991
NM_006037	B	OB	*HDAC4*	Histone deacetylase 4	3.272	8.475	2.468
NM_001742	B	OC	*CALCR*	Calcitonin receptor	-3.036	0.663	1.800
Not detected by microarray to be differentially regulated between females and males							
NM_004142	A, B, C	OB, OC, CB	*MMP25*	Matrix metalloproteinase 25	0.237	4.942	7.183

'Role' indicates the known or suspected role of the gene: A, angiogenic; B, osteogenic; and C, chondrogenic. 'Cell type' indicates the cell type that the gene is expressed in or affects: OB, osteoblast, OC, osteoclast, OS, osteocyte, or CB, chondroblast. 't OAF/OAM' is the t score of osteoarthritis (OA) female compared with OA male differential expression of gene: a postive value indicates upregulation in OA female and a negative one indicates downregulation in OA female. 't OA/CTL' is the t score of OA compared with CTL differential expression of gene: a positive value indicates upregulation in OA and a negative one indicates downregulation in OA. 't OA/OP' is the t score of OA compared with osteoporosis (OP) differential expression of gene: a positive value indicates upregulation in OA and a negative one indicates downregulation in OA. The moderated t-statistic score is based on the ratio of the log_2 _fold change to its standard error.

**Figure 2:**
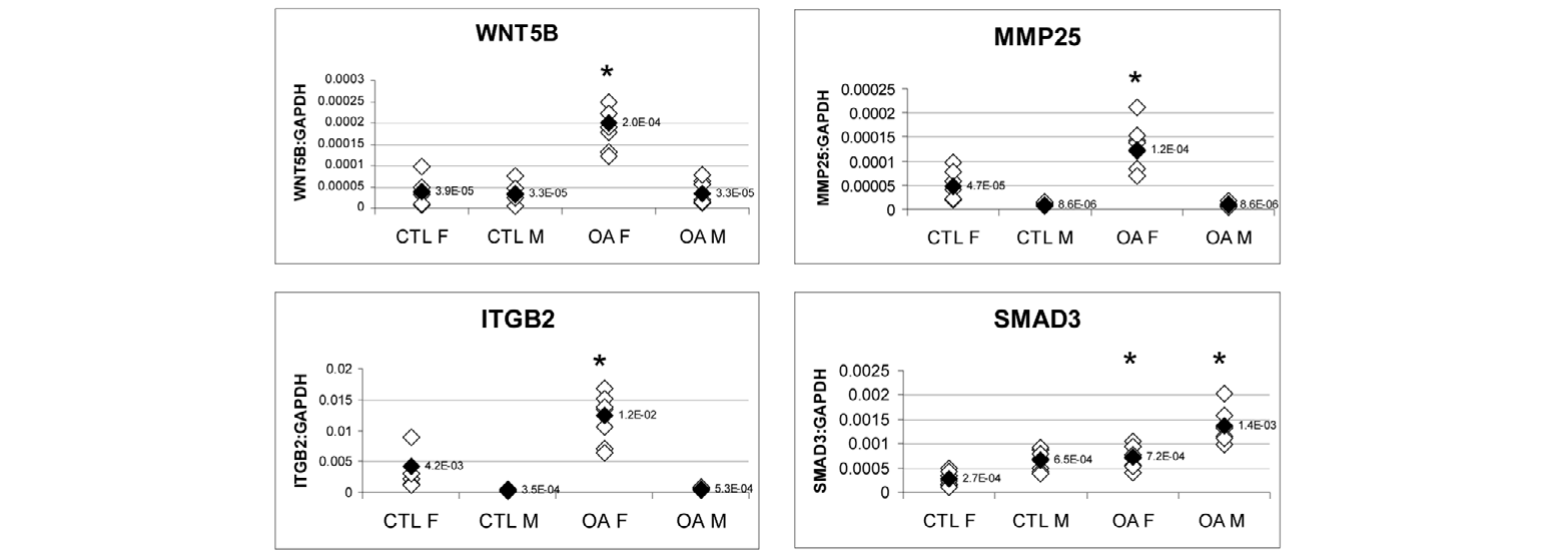
PCR analysis of *WNT5B*, *ITGB2*, *MMP25 *and *SMAD3 *expression between females and males in OA bone. For each gene, a graph depicts relative real-time PCR product/*GAPDH *cycle threshold (C_T_) ratios generated from osteoarthritis (OA) and control (CTL) female and male intertrochanteric bone samples analyzed. The mean of eight samples for each sample group analyzed is represented by black diamonds (mean values given alongside). Asterisks signify statistical significance (*P *< 0.01) for differential gene expression between OA and CTL bone. F, female; M, male.

The differential expressions of *WNT5B *and *ITGB2 *(along with *MMP25*) between females and males in OA bone were confirmed by real-time PCR (Figure 2). *MMP25 *was not originally identified as being differentially expressed between females and males in OA bone by microarray analysis. *WNT5B*, *ITGB2 *and *MMP25 *were all found to be differentially expressed only in females, and not in males, between OA and control bone. The OA/control ratios of expression for the *WNT5B *gene were 2.52 in female samples (*P *< 0.01) and 0.92 in male samples (*P *= 0.7486); those for the *ITGB2 *gene were 2.95 (*P *< 0.01) and 1.35 (*P *= 0.1173), respectively; and those for the *MMP25 *gene were 2.60 (*P *< 0.01) and 1.01 (*P *= 0.4748), respectively. There was also a significant difference in the total expression levels of these three genes between females and males, being approximately 6-fold, 14-fold and 23-fold higher for *WNT5B*, *MMP25 *and *ITGB2*, respectively, in females than in males in OA bone. Thirteen other genes (*ADAM8*, *ADM*, *ADAMTS4*, *ADFP*, *CD14*, *COL14A1*, *GADD45B*, *LRP1*, *S100A4*, *SMAD3*, *TGFBI*, *TIMP4 *and *TWIST1*) were also tested for differential expression between females and males in OA bone, but none of these was found to be differently expressed (data not shown). Genes were tested using female control samples 1 to 8 and OA samples 12 to 19 as compared with male control samples 37 to 44 (age range 60 to 85 years; mean [ ± SD] age 70.4 ± 7.3 years) and OA samples 47 to 54 (62 to 85 years; 70.3 ± 8 years; Table 1). The mean ages of the female and male OA groups did not significantly differ from those of the control groups. Of the other 13 genes examined by PCR, only *SMAD3 *had significant differences in total expression levels between males and females, being approximately 2-fold higher in males than in females (Figure 2). However, *SMAD3 *was similarly differentially regulated between OA and control bone in both females and males (ratio of OA/control: 2.64 in females [*P *< 0.01] and 2.08 in males [*P *< 0.01]).

## Discussion

In this study we identified altered expression of WNT and TGF-β/BMP signalling pathway component and target genes in OA bone distal to the disease site (from the IT region of the proximal femur). This was accomplished by using microarray analysis to compare gene expression in bone from individuals with end-stage OA disease and individuals without obvious OA (control or OP). The genes identified include those with known or suspected roles in osteoblast, osteocyte and osteoclast differentiation and function, as well as angiogenesis, suggesting perturbation of these processes and a role for altered bone remodelling in OA pathogenesis.

The trabecular bone sampled in this study included all of the cellular elements of bone, including the bone marrow. This has provided a 'snapshot' of the gene expression in OA bone, with contributions from all of the different cells in the bone microenvironment. Although the specific cell type(s) contributing to the altered gene expression cannot readily be identified, osteoblasts and osteocytes, which represent the most abundant cells in the trabecular bone [57], would be expected to contribute significantly to the altered gene expression measured. In addition, because we have analyzed bone from end-stage OA, it is difficult to determine unequivocally that the altered gene expression detected in the OA bone is causal or secondary to the disease. However, by sampling the IT region we have avoided secondary pathogenic changes that the subchondral bone undergoes at the joint as the disease progresses (such as sclerosis, osteophytes and cysts), which could confound identification of altered gene expression potentially responsible for the underlying subchondral bone remodelling. Therefore, we suggest that the altered gene expression identified in the IT region from OA bone may be informative about underlying systemic OA disease mechanisms that also operate at the joint in the subchondral bone.

The first main finding of this study is that many of the top-ranking differentially expressed genes in OA bone (Table 3) have known or suspected roles in osteoblast and osteocyte differentiation and function. (Also see Table 6 for descriptions of the functions of selected genes: *MEPE*, *IBSP*, *MT2A*, *ADM*, *STC1*, *IGFBP3*, *GADD45B*, *ADAMTS4*, *S100A4 *and *MMP25*.) Significantly, the changes in expression of these genes as a group suggest altered osteoblast and osteocyte activity in OA bone, which is consistent with increased bone volume fraction and under-mineralization previously reported in OA bone [3,4,11,22-25]. For instance, MEPE (matrix extracellular phosphoglycoprotein), which is highly expressed in osteoblasts and osteocytes, appears to be an important regulator of bone formation and mineralization. Targeted disruption of *MEPE *has been found to result in increased bone formation and bone mass [58]. *MEPE *was found to be downregulated in OA bone, which is consistent with the increased trabecular bone volume in OA. *MMP25 *(upregulated in OA bone) encodes a metalloproteinase that plays a role in MMP2 activation [59], and MMP2 is a major effector in osteocytes, with *MMP2*^-/- ^mice exhibiting disrupted osteocytic networks and altered bone remodelling and mineralization [60]. The *MMP2*^-/- ^mice have a complex bone phenotype that includes reduced bone volume in the long bones. Upregulation of *MMP25 *in OA bone is consistent with increased activity of MMP2 and increased bone volume.

**Table 6:** Bone related-functions of a selection of differentially expressed genes in osteoarthritis bone

Gene	Description/function	References
Bone remodeling, osteoblast: upregulated in osteoarthritis (OA) bone		
*S100A4*	Negative regulator of matrix mineralization in osteoblasts	[88,89]
*MMP25*	Metalloproteinase with role in matrix metalloproteinase (MMP)2 activation. Mice lacking MMP2 have disrupted osteocytes and altered bone mineralization	[59,60]
Bone remodeling, osteoblast: downregulated in OA bone		
*MEPE*	Extracellular matrix protein, highly expressed in osteocytes	[58]
*IBSP*	Major constituent of the bone matrix, thought to initiate and regulate mineralization	[90]
*MT2A*	Metallothionein proteins (also MT1L and MT1G) have roles in regulating osteoblast differentiation and mineralization	[91,92]
*ADM*	Adrenomedullin stimulates osteoblast activity, but also interacts with and influences the effects of key bone regulators insulin-like growth factor (IGF)1 and transforming growth factor (TGF)-β	[93,94]
*STC1*	Stanniocalcin inhibits calcium uptake and has inhibitory effect on bone growth during development	[95,96]
*IGFBP3*	Constitutive over-expression of IGF1-binding protein (IGFBP)3 impairs osteoblast proliferation and bone formation	[97]
*GADD45B*	Mice deficient in GADD45B protein have defective bone mineralization	[98]
*ADAMTS4*	Metalloproteinase involved in remodelling extracellular matrix. Upregulated in fracture healing. Expressed in osteocytes and osteoblasts	[99,100]
*LTF*	Lactotransferrin is an anabolic bone factor	[101]
*DF*	Adipsin inhibits osteoblastogenesis	[102]
Bone remodeling, osteoclast: upregulated in OA bone		
*ADAM8*	Stimulatory role in osteoclast formation and differentiation	[103]
*CCR2*	Receptor for CC chemokine ligand (CCL)2, which promotes recruitment and fusion of monocytes/osteoclast precursors	[104]
*RAC2*	Member of the Rho-GTPase subfamily. Involved in organisation of cytoskeleton and adhesion of osteoclasts to bone	[105]
*CD14*	Monocyte/osteoclast precursor marker. CD14-deficient mice have increased bone mass	[106,107]
*ANXA2*	Stimulates osteoclast precursor proliferation and differentiation through production of granulocyte-macrophage colony-stimulating factor (GMCSF) and receptor activator of nuclear factor-kB ligand (RANKL)	[108]
*GSN*	Gelsolin deficiency in mice blocks podosome assembly in osteoclasts and produces increased bone mass	[109]
*ITGB2*	Adhesion molecule important in cell-to-cell contacts during the early stage of osteoclast development	[110,111]
Bone remodeling, osteoclast: downregulated in OA bone		
*IGFBP3*	Over-expression of IGFBP3 in mice increases osteoclast number and bone resorption	[97]
*STC1*	Stanniocalcin suppresses osteoclast activity	[96]
*PTEN*	Over-expression of PTEN suppresses RANKL-stimulated signal transduction during osteoclast differentiation	[112]
*FOXP1*	Transcriptional repressor that has role in modulating monocyte differentiation	[113]
*PDE4A*	Down-regulated during monocyte to macrophage/osteoclast differentiation	[114]
WNT pathway components and modulators: upregulated in OA bone		
*WNT5B*	WNT ligand with roles in osteoblastogenic and chondrogenic differentiation	[68,69,70]
*CTNNB1*	Mice lacking b-catenin in osteoblasts develop severe osteopenia with increased osteoclastogenesis and impaired osteoblastogenesis	[115]
*AKT3*	Member of the AKT kinase family. Role in regulating osteoblast lifespan	[116,117]
*NHERF1*	Mediates parathyroid hormone receptor signalling. Interacts with b-catenin, potentiating the effects of parathyroid hormone (PTH) on WNT signalling in bone.	[118,119]
WNT pathway components and modulators: downregulated in OA bone		
*FZD3*	WNT5B co-receptor	[120]
*PTEN*	Modulates/antagonises WNT signalling. Roles in osteoclast and osteoblast differentiation	[112,117]
*APC*	Loss of APC in mice leads to increased bone mass	[115]
*AXIN2*	Negatively regulates both expansion of osteoprogenitors and maturation of osteoblasts through its modulation of WNT signalling	[121]
TGF-β/bone morphogenic protein (BMP) pathway components and modulators: upregulated in OA bone		
*ACVR1*	BMP and activin A receptor. Mutation in receptor causes ectopic osteogenesis	[122]
*SMAD3*	Important mediator of TGF-β signalling and regulator of osteoblastogenesis and bone formation	[80,82]
*ID1*	Transcription factor with roles in bone formation and osteoblast proliferation and differentiation	[123]
*RUNX2*	Key transcription factor involved in promoting osteoblast differentiation	[76]
TGF-β/BMP pathway components and modulators: downregulated in OA bone		
*TGFB1*	Growth factor with key role in regulating bone development and metabolism	[34,80]
*INHBA*	TGF-β family member. Can act as either inhibitor or activator of bone formation and osteoblast differentiation	[124,125]
*BMP5*	Secreted signalling molecule involved in skeletal development and genetically implicated in OA	[29]
*FST*	TGF-β/BMP antagonist that inhibits osteoblast differentiation	[126]

Interestingly, and in contrast to the large number of differentially expressed genes identified in OA bone with osteoblast-related and osteocyte-related roles, a substantial group of top-ranking differentially expressed genes identified in OP bone (data not shown) have known or suspected roles in osteoclast-lineage cells. The change in expression of these genes as a group is consistent with increased osteoclast numbers, activity and bone resorption, leading to the reduction of bone volume seen in OP. There was also a group of differentially expressed genes identified in OA bone that have known or potential roles in osteoclast function (Tables 3 to 5). However, the overall change in expression of these genes, as a group, was not as consistent as for those genes identified with osteoblast-related functions. For instance, the change in expression of a subset of these genes (*ADAMTS4*, *GADD45B*, *IGFBP3 *and *CSTA*) is consistent with decreased osteoclast activity and increased bone volume in OA, whereas the change in expression of another subset of these osteoclast-related genes (*ADAM8*, *STC1*, *CCR2*, *RAC2*, *CRYAB*, *CYP1B*, *CD14*, *PTEN*, *ANXA2 *and *GSN*) suggests upregulated osteoclast activity (Table 6). It is now becoming clear that, in addition to osteoblasts being intimately involved in influencing osteoclast-lineage cell differentiation and function [61], the converse may also be true [62,63]. As a result, perturbed osteoblast or osteoclast gene expression could lead to complex changes in communication between these bone cell types and their remodelling behaviour in OA bone. Therefore, differential expression of a subset of the osteoclast-related genes, suggesting upregulated osteoclast activity, is consistent with the increased levels of bone remodelling seen in OA bone and perhaps a net gain of under-mineralized bone rather than the net loss of bone volume seen in OP.

Important inter-relationships between bone remodelling and angiogenesis are also now emerging, and so perturbations to angiogenic molecular pathways could contribute to the changes seen in OA bone. Consistent with a role for increased angiogenesis in OA bone, leading to increased bone volume and potentially turnover, is the upregulation of a group of top-ranking differentially expressed genes with recognized pro-angiogenic functions (*MMP25*, *S100A4*, *FOSB*, *TFF3*, *CTSG *and *LTB*) and downregulation of a group of genes that negatively influence angiogenesis (*HIG2*, *ADAMTS4*, *ANGPTL4*, *STC1*, *KLF6*, *IGFBP3*, *TIMP4 *and *GDF15*).

In addition, a subset of the genes with roles in osteoblasts, particularly those that encode secreted, cell surface and extracellular matrix molecules, including *ADM*, *IBSP*, *MMP25*, *STC1*, *IGFBP3*, *WNT5B*, *FST*, *TGFB1*, *BMP5 *and *INHBA*, also have potential chondrogenic functions. Altered expression of many of these genes is consistent with the proposal that similar altered expression of these genes in osteoblasts in the subchondral bone microenvironment could interfere with chondrocyte or cartilage metabolism. For instance, *ADM*, which is downregulated in OA bone has a pro-chondrogenic role [64]. Reduced levels of ADM could negatively affect chondrocyte/cartilage metabolism.

The second significant and connected observation from this study was that many of the top-ranking differentially expressed genes identified in OA bone (with osteoblast, osteocyte and osteoclast related roles) were WNT or TGF-β/BMP signalling pathway target genes. This observation, on closer examination of the ranked list of differentially expressed in OA bone, led to the identification of additional sets of differentially expressed genes that were WNT or TGF-β/BMP signalling pathway component or modulator genes. These data together suggest that the WNT and TGF-β/BMP signalling pathways are altered in OA bone and may play a role(s) in OA pathogenesis. Both the WNT and TGF-β/BMP signalling pathways have been implicated in influencing bone mass and bone remodelling [32-34] and have been demonstrated to do this by controlling both osteoblast and osteoclast differentiation and function [65-67].

WNT signalling, in terms of bone mass and bone remodelling, is a very complex process that depends on the interplay of a large number of WNT ligands, the receptors they complex with, prevailing antagonists and particular combinations of β-catenin/transcription factor complexes that ultimately control the expression of the target genes. Interestingly, the only gene encoding a WNT ligand, *WNT5B*, that was identified as being differentially expressed (upregulated in OA bone) in the present study was recently demonstrated to increase in expression during *in vitro *osteoblast differentiation [68]. The protein encoded by *WNT5B *is known to have both stimulatory and inhibitory effects on bone and cartilage cells, and signals through both the canonical and noncanonical WNT signalling pathways, depending on the receptor it complexes with at the cell surface [68-70]. Along with *WNT5B*, there were also several other important WNT pathway related genes that were altered in their expression in OA bone. Genes for the *WNT5B *co-receptor *FZD3 *and extracellular WNT antagonist *SFRP5 *were under-expressed, relative to controls, suggesting increased WNT signalling. Downregulation of the intracellular signalling cascade genes *PTEN*, *APC *and *AXIN2*, and upregulation of *CTNNB1*, *AKT3 *and *NHERF1 *are also consistent with increased WNT signalling. *CTNNB1 *encodes β-catenin, which is the central downstream mediator of canonical WNT signalling, which forms a complex with lymphoid enhancer factor/T cell factor (LEF/TCF) transcription factors to modulate target gene expression [71], whereas *PTEN*, *APC*, *AXIN2*, *AKT3 *and *NHERF1 *gene products modulate β-catenin activity (Table 6).

Like WNT signalling, TGF-β/BMP signalling is similarly complex, with a large family of ligands, cognate receptors and intracellular signalling molecules involved in the pathway, exerting both stimulatory and inhibitory effects on bone remodelling. Several TGF-β/BMP signalling pathway component and modulator genes that influence osteoblast function, bone remodelling and bone mineralization were identified as altered in OA bone. These included *TGFB1*, *INHBA*, *ACVR1*, *BMP5*, *FST *and *SMAD3 *(Table 6).

There is significant crosstalk between the WNT and TGF-β/BMP signalling pathways. β-Catenin, SMAD3 and runt-related transcription factor (RUNX)2 potentially play important roles in mediating the crosstalk between the WNT and TGF-β/BMP signalling pathways via direct interactions and in complexes with the TCF/LEF transcription factor family members in the nucleus [72-75]. We observed increased *RUNX2 *expression in OA bone in this study, which is consistent with increased osteoblast differentiation and activity in OA bone. *RUNX2*, which is a WNT inducible gene, encodes a transcription factor that plays roles in mediating both WNT and TGF-β/BMP signalling, and is essential for osteoblast differentiation and skeletal development [66,75-77]. Intriguingly, decreased expression of *RUNX2 *has been shown to reduce cartilage destruction and subchondral bone changes in a mouse joint instability OA model [78], suggesting a role for increased *RUNX2 *expression in OA pathogenesis. An important role for *RUNX2 *in OA pathogenesis is supported by our microarray data. The products of several of the top-ranking differentially expressed genes identified, such as TWIST1 (twist homologue 1) [79], FOXF1 (forkhead box F1), ID1 (inhibitor of DNA binding 1), HDAC4 (histone deacetylase 4) and SMAD3, modify *RUNX2 *expression or interact with and modify RUNX2 function. SMAD3 is an important mediator of TGF-β regulation of bone mechanical properties and composition [80]. TGF-β represses *RUNX2*, and one of the ways it does this is through recruitment of the histone deacetylase HDAC4 by SMAD3 [81]. However, *SMAD3 *over-expression (*SMAD3 *was upregulated in OA bone in the present study) has also been reported to induce *RUNX2 *expression and osteoblast differentiation [82]. Significantly, several of the highest ranking differentially expressed genes identified in this study in OA bone are targets of RUNX2 and SMAD3, such as *GADD45B *[83], *ADAMTS4 *[84] and *MEPE *[82].

Finally, the molecular mechanisms that are responsible for the greater incidence of OA in females are not known. Genes may operate differently in the two sexes, at different body sites and on different disease features within body sites [27]. Interestingly, of the relatively small number of differences between females and males in OA bone identified in this study, there were significant numbers of genes that were involved in both osteoclast (for example, *ANXA2*, *GSN*, *ITGB2*, *FOXP1 *and *PDE4A*) and osteoblast (*LTF*, *DF *and *TGFB1*) function and hence bone remodelling (Tables 5 and 6). Collectively, the differential expression of these genes is consistent with increased bone turnover in OA females compared with males, suggesting an OA disease mechanism and perhaps partly accounting for a greater incidence of OA in females than in males.

A number of the highest ranking differentially expressed genes between OA females and males include WNT signalling pathway components such as *WNT5B *and the transcription factor genes *EAF2 *and *CTBP2*. In addition *MMP25 *and *ITGB2 *are WNT target genes, suggesting a difference between females and males in WNT signalling that may have an impact on the OA bone microenvironment. There is also evidence of crosstalk between WNT and oestrogen signalling pathways via functional interaction between β-catenin and oestrogen receptor-α [85]. A number of the genes identified in our study, including *WNT5B*, *ITGB2*, *GSN*, *VCAM1*, *LTF *and *DF*, are affected by oestrogen, potentially providing a mechanism by which they are differentially expressed in females compared with males. Examples of sexual dimorphism in mammalian gene expression related to different responses to disease by females and males are beginning to be identified [86]. The differences in expression levels of *WNT5B*, *ITGB2 *and *MMP25 *detected between females and males in OA bone is of interest and marks these genes as good candidates for further investigation into the sex disparity in OA.

In this study we observed small gene expression ratios in both microarray and real-time PCR analyses. These are probably contributed by the complex mix of cells being assayed, along with the subtle changes to bone that are observed in OA distal to the affected joint, and the often slow, age-dependent onset of the disease. Furthermore, OA is a multifactorial, multigene disorder (and perhaps even a heterogenous group of disorders that lead to similar bone changes, cartilage degeneration and ultimately loss of joint function). Therefore, it is to be expected that many genes and small changes in the expression of these genes would be involved in OA pathogenesis. Microarray analysis is able to reliably detect small (<2-fold) changes that prove to be biologically relevant [87], and in our study we were able to confirm the large majority of the differentially expressed genes by real-time PCR analysis. Furthermore, the power of the microarray analysis approach lies in its ability to detect genome-wide, coordinated, or similarly regulated differential gene expression, pointing to perturbed signalling pathways and importantly downstream molecular processes. Our study has identified such relationships between commonly regulated target genes (via WNT and TGF-β/BMP signalling pathways) that play roles, in particular, in osteoblasts, osteocytes and osteoclasts, potentially influencing bone formation, mineralization and remodelling.

## Conclusion

In conclusion, we identified altered gene expression in bone from individuals with primary hip OA at a site distal to the diseased joint. This information is of interest because it identifies genes that potentially play roles in systemic physiological bone turnover or in skeletal disease processes, and implicate altered WNT and TGF-β/BMP signalling in OA pathogenesis. Further work sampling from individuals with early OA will be required to determine whether the genes identified as differentially expressed in OA bone are causal or secondary to the altered bone seen in OA.

## Abbreviations

AMF = Adelaide Microarray facility; BMP = bone morphogenic protein; C_T _= cycle threshold; IL = interleukin; IT = intertrochanteric; LEF = lymphoid enhancer factor; LIMMA = linear models for microarray analysis; MMP = matrix metalloproteinase; OA = osteoarthritis; OP = osteoporosis; PCR = polymerase chain reaction; RUNX = runt-related transcription factor; SD = standard deviation; TCF = T-cell factor; TGF = transforming growth factor; WNT = wingless MMTV integration.

## Competing interests

The authors declare that they have no competing interests.

## Authors' contributions

NLF and DMF conceived the study. BH contributed to study design and performed the acquisition of the microarray and real-time PCR data. AT advised on the microarray experiment design and performed the statistical analyses of the microarray data. BH and AT analyzed and interpreted the data. BH, DMF and NLF prepared the manuscript. All authors read and approved the final manuscript.
